# The Epidemiology of Inflammatory Bowel Disease in Oceania: A Systematic Review and Meta-Analysis of Incidence and Prevalence

**DOI:** 10.1093/ibd/izad295

**Published:** 2023-12-30

**Authors:** Angela J Forbes, Chris M A Frampton, Andrew S Day, Gilaad G Kaplan, Richard B Gearry

**Affiliations:** Department of Medicine, University of Otago Christchurch, Christchurch, New Zealand; Department of Medicine, University of Otago Christchurch, Christchurch, New Zealand; Department of Paediatrics, University of Otago Christchurch, Christchurch, New Zealand; Department of Medicine, Cumming School of Medicine, University of Calgary, Calgary, Alberta, Canada; Department of Community Health Sciences, Cumming School of Medicine, University of Calgary, Calgary, Alberta, Canada; Department of Medicine, University of Otago Christchurch, Christchurch, New Zealand

**Keywords:** Australia, New Zealand, Oceania, epidemiology, incidence, prevalence

## Abstract

**Background:**

Past studies have shown high rates of inflammatory bowel disease (IBD) in Australia and New Zealand (NZ). We aimed to describe the epidemiology of IBD in Australia, NZ, and the surrounding region (collectively termed Oceania) by conducting a systematic review and meta-analysis.

**Methods:**

Electronic databases were searched from inception to April 2023 for studies reporting incidence or prevalence rates of IBD, Crohn’s disease (CD), or ulcerative colitis (UC) in Oceania. All study designs were included. A meta-analysis calculated pooled estimates of incidence and prevalence, and a sensitivity analysis compared the pooled population-based studies with the non–population-based studies and the Australian and NZ studies separately.

**Results:**

Nineteen incidence and 11 prevalence studies were included; 2 studies were from the Pacific Islands, with the rest coming from Australia and NZ. Pooled estimates showed high incidence rates of 19.8 (95% confidence interval [CI], 15.8-23.7) for IBD, 8.3 (95% CI, 6.9-9.8) for CD, and 7.4 (95% CI, 5.7-9.1) for CD per 100 000 person-years. CD was more common than UC in most studies. The pooled estimates for the prevalence studies were 303.3 (95% CI, 128.1-478.4) for IBD, 149.8 (95% CI, 71.0-228.5) for CD, and 142.2 (95% CI, 63.1-221.4) for UC per 100 000 persons. Studies using population-based data collection methods showed higher pooled rates for both incidence and prevalence.

**Conclusions:**

The incidence and prevalence of IBD in Oceania is high. The studies were heterogeneous and there were several geographic areas with no information, highlighting the need for more epidemiological studies of IBD.

**Key Messages**

**What is already known?**

Inflammatory bowel diseases (IBDs) impact patients’ quality of life and can burden health systems through medications, hospitalizations, and surgery costs.

**What is new here?**

High rates of IBD were observed in New Zealand and Australian studies, and only limited information was available from the Pacific Islands.

**How can this study help patient care?**

The incidence and prevalence rates of IBD observed in this study can be used to support health service planning and enable predictions of future disease burden in Oceania.

## Introduction

Inflammatory bowel diseases (IBDs) are chronic gastrointestinal conditions treated with medications, surgery, and hospitalization, which impact patients’ lives and health systems.^1-3^ Understanding the epidemiology of IBD enables the estimation of current and future burden of Crohn’s disease (CD), ulcerative colitis (UC), and IBD unclassified (IBDU).^4^

Recent international studies have found increasing prevalence but stable incidence for IBD in Western countries^5^ but rising incidence rates in newly Westernized countries in areas such as Asia^6^ and Latin America.^7^ A 4-stage epidemiological model of IBD predicts that periods of high incidence will be followed by a compounding prevalence stage in which incidence plateaus as a country develops but prevalence rates continue to grow.^8^

Oceania is a diverse region of the South Pacific consisting of the Westernized countries Australia and New Zealand and the regions of Micronesia, Melanesia, and Polynesia that are comprised of many less developed and smaller island nations.^9^ All countries in Oceania have a mix of indigenous peoples and migrants, and this genetic diversity, along with the environmental differences between countries, make it a good region in which to study IBD epidemiology.

High incidence rates of IBD have been reported in New Zealand, including one of the highest rates of CD in the world from a 2004 population-based study in the Canterbury region.^10^ Subsequent Australian studies^11-13^ using the same methodology have also reported rates of IBD incidence and prevalence in line with those observed in North America and Northern Europe.^14^ Population-based data collection approaches have the advantage of minimizing selection bias toward more severe presentations, which may occur in hospital-based studies.^15^ But in areas of limited research, restricting analysis to only population-based methods significantly reduces the number of available studies, as seen in Ng et al,^14^ who included only studies from Melbourne and Canterbury in their worldwide review of IBD. Consequently, to get an in-depth understanding of IBD epidemiology across Oceania, this study aimed to conduct the first systematic review and meta-analysis of incidence and prevalence in this region. This work also gathered information on the characteristics of patients within these cohorts to compare attributes, such as phenotype, between studies.

## Methods

### Search Strategy

A systematic literature review of the electronic databases MEDLINE, EMBASE, Scopus, and Web of Science from inception to April 2023 was conducted using the PRISMA (Preferred Reporting Items for Systematic Reviews and Meta-Analyses) guidelines (Supplementary Table 1).^16^ No limits were placed on language or study design, and reference lists were checked for additional publications.

### Selection Criteria

All studies from Oceania (Supplementary Table 2) reporting either incidence or prevalence rates of IBD, CD, or UC were eligible to be included. Genetic studies and etiology-based research were excluded, as were review articles describing studies already included individually. Studies reporting on only pediatric cohorts were excluded, as they have lower incidence and prevalence rates, and these have been described in another systematic review and meta-analysis.^17^ Two authors (A.J.F. and C.M.A.F.) screened article titles and abstracts and then accessed the full text of articles potentially meeting the inclusion criteria. Where there was any doubt about the inclusion or exclusion of a study, a consensus was reached with the other authors.

### Data Extraction

Crude annual incidence and prevalence rates per 100 000 person-years/persons along with 95% confidence intervals (CIs) and information on study design, year, and location were extracted into a template. Population-based studies were defined as those capturing the entire population of a defined area.^1^ Additional study characteristics including the average age, sex, ethnicity, phenotype, surgery, and family history of IBD of participants were recorded in a second template. The risk of selection and misclassification bias was evaluated for each study using a modified version of the Newcastle-Ottawa quality assessment scale.^18^ Earlier studies did not have full access to colonoscopy and imaging technologies, but the term standard diagnostic criteria was assigned to the studies describing a diagnosis from a gastroenterologist that used a combination of clinical symptoms and laboratory, radiological, endoscopic, and histologic findings.

### Data Synthesis

Where studies reported incidence rates for time periods other than 1 year, these were converted to average annual rates. CD:UC ratios were calculated by dividing the number of patients with CD by the number of patients with UC, with values above 1 indicating a predominance of CD. When studies did not publish 95% CIs for estimates, these were derived using the information provided. Data synthesis used Microsoft Excel.

A random-effects model was used to calculate the pooled estimates, and *I*^2^ was used to assess heterogeneity. A sensitivity analysis calculated pooled estimates for the population-based studies to evaluate the impact of study design on the *I*^2^ values and the observed incidence and prevalence rates.

### Ethical Considerations

No ethical approval was required for this systematic review and meta-analysis, as data were extracted from published sources.

## Results

### Search Results

**Figure 1.**
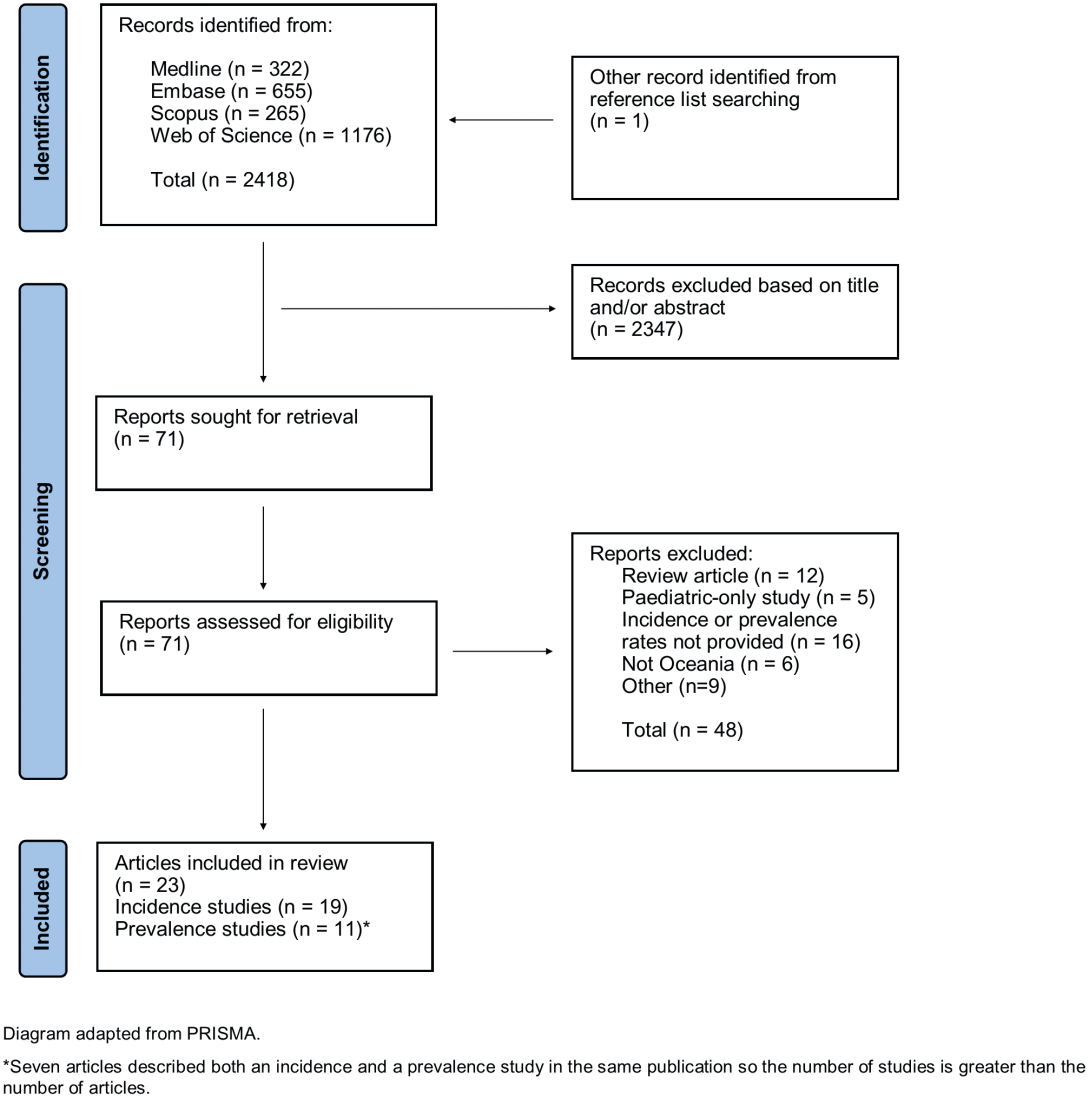
Study selection process. PRISMA, Preferred Reporting Items for Systematic Reviews and Meta-Analyses.

The systematic literature search identified 2418 articles, and a further article was identified from reference list searching; 71 of these were selected for full-text review (Figure 1). Twenty-three articles met the inclusion criteria, with 7 of these describing both an incidence and a prevalence study within the same publication. From these articles, 30 study estimates of either incidence or prevalence were selected for full analysis. These estimates derived from Australia (n = 15), New Zealand (n = 13), Fiji (n = 1), and French Polynesia (n = 1). Study dates ranged from 1954 to 2020 (Table 1). The highest incidence rate for IBD was 39.8 per 100 000 person-years from Canterbury, New Zealand, in 2014,^22^ and the lowest rate was 1.9 per 100 000 person-years from a 1985 to 1986 study from Fiji.^29^ For prevalence, the highest rate of IBD was 653 per 100 000 persons from a 2019 study in Australia,^34^ and the lowest rate was 17 per 100 000 persons from a 2018 study in French Polynesia.^35^

**Table 1.** All included studies incidence and prevalence rates.

First author, publication year	Country, area	Study period	Study design	CD:UC ratio	IBD rate (95% CI)	CD rate (95% CI)	UC rate (95% CI)	IBDU rate (95% CI)
Incidence								
Qiu et al, 2022^19^	New Zealand, Rotorua	2001-2020	Hospital-based incidence	1.2^a^	7.6 (6.5 to 8.9)^a^	4.0 (3.2 to 5.0)^a^	3.4 (2.7 to 4.3)^a^	0.2 (0.1 to 0.5)^a^
Seleq et al, 2023^20^	New Zealand, Waikato	2010-2019	Population-based incidence	0.8^a^	18.1 (16.8 to 19.4)^a^	7.9 (7.0 to 8.8)^a^	9.7 (8.7 to 10.7)^a^	0.5 (0.3 to 0.8)^a^
Flanagan et al, 2022^21^	Australia, Mackay, Isaac, Whitsunday	2017-2018	Population-based incidence	0.8^a^	32.2 (24.8 to 41.8)	13.2 (8.8 to 19.9)	17.3 (12.1 to 24.7)	1.7 (0.6 to 5.4)
Su et al, 2016^22^	New Zealand, Canterbury	2014	Population-based incidence	1.9^a^	39.8 (34.4 to 45.3)	26.0 (21.6 to 30.4)	13.4 (10.2 to 16.6)	0.4 (−0.2 to 0.9)
Bhatia et al, 2019^13^	Australia, Tasmania	2013-2014	Population-based incidence	1.2^a^	29.0 (24.4 to 33.7)	14.4 (11.1 to 17.7)	12.3 (9.2 to 15.3)	2.4 (1.0 to 3.7)
Iyngkaran et al, 2015^23^	Australia, Darwin	2013	Hospital-based incidence	1.1^a^	19.6 (14.1 to 28.9)	10.1 (6.1 to 16.8)	9.5 (5.6 to 15.9)	—
Coppell et al, 2018^24^	New Zealand, Otago	1996-2013	Hospital-based incidence	1.3^a^	14.1 (12.9 to 15.4)^a^	7.6 (6.7 to 8.6)^a^	5.4 (4.7 to 6.2)^a^	1.1 (0.8 to 1.5)^a^
Niewiadomski et al, 2015^25^	Australia, Barwon	2010-2013 2007-2008	Population-based incidence	1.5^a^	21.5 (18.9 to 24.3)^a^	12.4 (10.5 to 14.6)^a^	8.2 (6.7 to 9.9)^a^	0.9 (0.4 to 1.5)^a^
Day et al, 2014^26^	New Zealand, Nelson	2012	Population-based incidence	—	—	15.2 (8.8 to 24.5)^a^	—	—
Studd et al, 2016^12^	Australia, Barwon	2010-2011	Population-based incidence	2.0^a^	24.2 (18.9 to 30.5)	14.7 (10.6 to 19.7)	7.5 (4.7 to 11.4)	2.0 (0.8 to 4.5)
Wilson et al, 2010^11^	Australia, Barwon	2007-2008	Population-based incidence	1.6^a^	29.3 (23.5 to 36.7)	17.4 (13.0 to 23.2)	11.2 (7.8 to 16.1)	0.8 (0.2 to 2.8)
Hanigan et al, 2008^27^	Australia, North Brisbane	2005	Hospital-based incidence	—	30.3 (26.5 to 34.5)^a^	—	—	—
Gearry et al, 2006^10^	New Zealand, Canterbury	2004	Population-based incidence	2.2^a^	25.2 (20.8 to 30.2)	16.5 (13.0 to 20.4)	7.6 (5.3 to 10.6)	1.1 (0.1 to 2.0)
Anseline, 1995^28^	Australia, Hunter Valley	1967-1991	Hospital-based incidence	—	—	1.7 (1.4 to 2.0)^a^	—	—
Probert et al, 1991^29^	Fiji	1985-1986	Hospital-based incidence	0.1^a^	1.9 (1.2 to 3.4)^a^	0.1 (0.0 to 0.5)^a^	1.7 (0.3 to 3.1)^a^	—
McDermott et al, 1987^30^	Australia, Melbourne	1980-1981	Hospital-based incidence	0.5^a^	2.7 (2.2 to 3.3)^a^	0.8 (0.5 to 1.2)^a^	1.5 (1.1 to 2.0)^a^	0.3 (0.1 to 0.5)^a^
Schlup et al, 1986^31^	New Zealand, Dunedin	1972-1981	Hospital-based incidence	—	—	2.4 (1.8 to 3.2)^a^	—	—
Eason et al, 1982^32^	New Zealand, Auckland	1969-1978	Hospital-based incidence	0.3^a^	7.4 (6.8 to 8.0)^a^	1.8 (1.5 to 2.1)^a^	5.5 (5.0 to 6.0)^a^	—
Wigley et al, 1962^33^	New Zealand, Wellington	1954-1958	Hospital-based incidence	—	—	—	5.5 (4.1 to 7.2)^a^	—
Prevalence								
Qiu et al, 2022^19^	New Zealand, Rotorua	2020	Hospital-based prevalence	1.3^a^	169.3 (146.9 to 194.2)^a^	85.9 (70.3 to 104.0)^a^	66.2 (52.6 to 82.3)^a^	17.2 (10.8 to 26.1)^a^
Seleq et al, 2023^20^	New Zealand, Waikato	2019	Population-based prevalence	0.8^a^	375.6 (357.6 to 394.3)^a^	164.6 (152.8 to 177.1)^a^	203.5 (190.4 to 217.4)^a^	7.5 (5.2 to 10.4)^a^
Busingye et al, 2021^34^	Australia	2019	General practice-based prevalence	0.9^a^	653.0 (623.0 to 684.0)	306.0 (292.0 to 321.0)	334.0 (317.0 to 352.0)	12.0 (10.7 to 13.4)^a^
Grymonpré et al, 2019^35^	French Polynesia	2018	Hospital-based prevalence	0.6^a^	17.0 (12.7 to 22.3)^a^	6.0 (3.6 to 9.4)^a^	11.0 (7.6 to 15.5)^a^	0.7 (0.1 to 2.4)^a^
Pudipeddi et al, 2021^36^	Australia, Sydney	2016	Population-based prevalence	1.0^a^	414.0 (371.0 to 456.0)	186.0 (158.0 to 215.0)	182.0 (154.0 to 210.0)	45.0 (31.0 to 60.0)
Bhatia et al, 2019^13^	Australia, Tasmania	2014	Population-based prevalence	1.1^a^	335.0 (319.2 to 350.9)	170.3 (159.0 to 181.6)	156.5 (145.7 to 167.3)	8.2 (5.7 to 10.7)
Iyngkaran et al, 2015^23^	Australia, Darwin	2014	Hospital-based prevalence	1.1^a^	131.2 (116.5 to 147.3)^a^	67.0 (56.5 to 78.5)^a^	62.0 (52.1 to 73.4)^a^	2.0 (0.9 to 5.2)^a^
Day et al, 2014^26^	New Zealand, Nelson	2012	Population-based prevalence	1.4^a^	389.0 (351.5 to 429.4)^a^	226.9 (198.6 to 258.1)^a^	162.1 (138.4 to 188.7)^a^	—
Studd et al, 2016^12^	Australia, Barwon	2011	Population-based prevalence	1.5^a^	344.6 (309.6 to 383.4)	197.3 (170.5 to 226.5)	136.0 (114.1 to 160.9)	8.5 (4.1 to 17.1)
Gearry et al, 2006^10^	New Zealand, Canterbury	2005	Population-based prevalence	1.1^a^	308.2 (292.2 to 324.3)	155.2 (143.8 to 166.6)	145.0 (134.0 to 156.0)	8.0 (5.4 to 10.6)
Selinger et al, 2013^37^	Australia, Sydney	1992	Population-based prevalence	0.8^a^	199.4 (187.3 to 212.1)^a^	83.4 (75.7 to 91.7)^a^	106.6 (97.8 to 115.9)^a^	9.4 (7.0 to 12.4)^a^

Crude incidence rates per 100 000 person-years, crude prevalence rates per 100 000 population. Coppell et al^24^ reported only age-standardized rates. Probert et al’s^29^ rates are for Fijian Indians who made up 87% of the IBD cases recorded. Iyngkaran et al’s^23^ incidence rates for the nonindigenous population (97% of cases).

Abbreviations: CD, Crohn’s disease; CI, confidence interval; IBD, inflammatory bowel disease; IBDU, inflammatory bowel disease unclassified; UC, ulcerative colitis.

^a^Data derived from supporting information by the current authors.

## Incidence Studies

**Figure 2.**
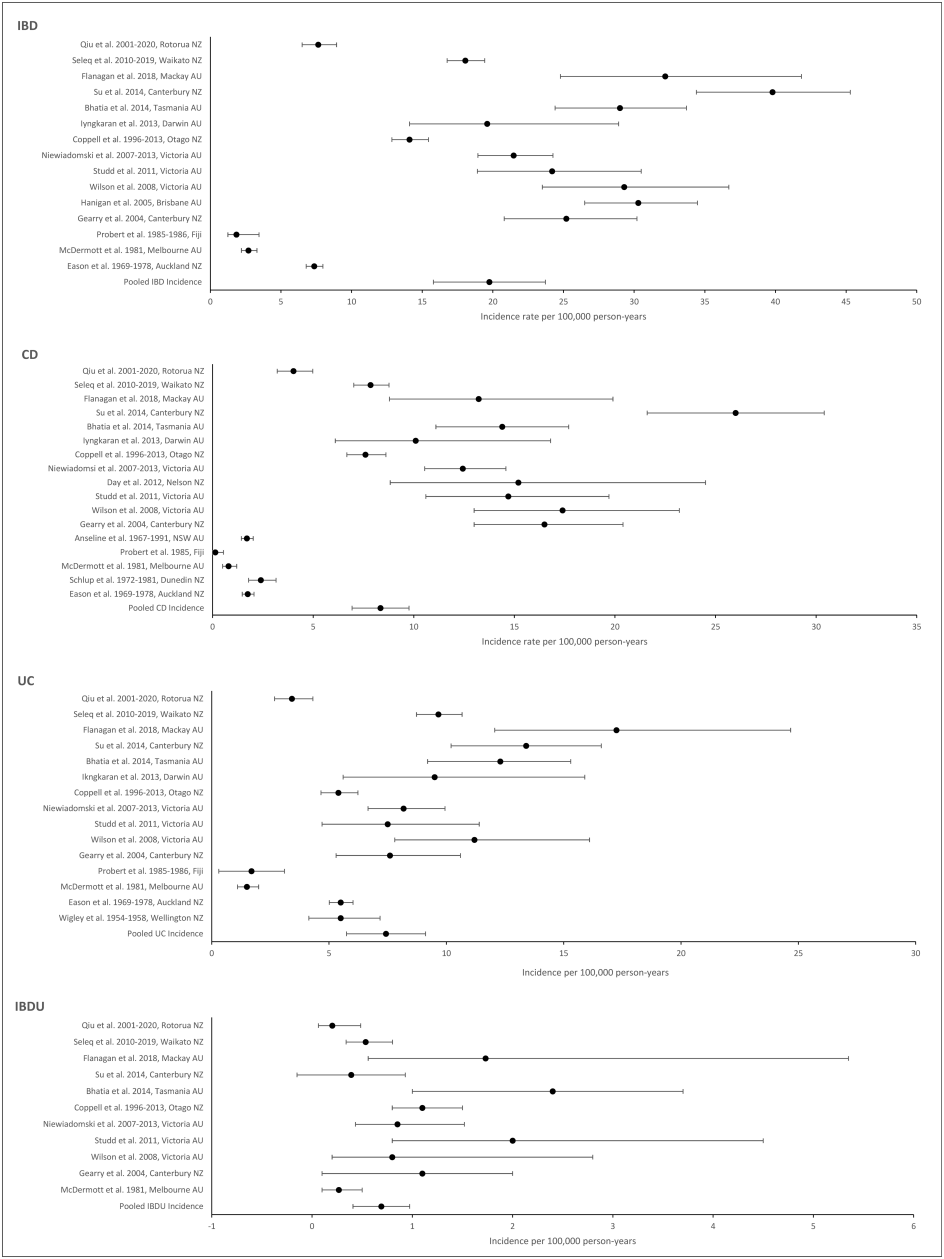
Inflammatory bowel disease (IBD), Crohn’s disease (CD), ulcerative colitis (UC), and IBD unclassified (IBDU) incidence rates. AU, Australia; NZ, New Zealand.

Nineteen studies reported incidence rates, 2 of these studies focused only on CD,^26,28,31^ and 1 study reported only on UC.^33^ Nine studies were from areas within New Zealand, 9 were from areas within Australia, and 1 study was from Fiji (Figure 2).

Six of the incidence studies collected data for 10 years or more,^19,20,24,28,31,32^ enabling short-term changes in incidence rates to be averaged over time. These longer-running studies showed similar incidence rates to the shorter-length studies.

Population-based data collection methods were used for 9 studies,^10-13,20-22,25,26^ while 10 used hospital-based methods.^19,23,24,27-33^ All of the earlier studies (pre-2000)^28-33^ used a hospital-based approach. These earlier studies each showed incidence rates of <8 per 100 000 person-years (Supplementary Figure 1).

The highest incidence rate was 39.8 per 100 000 person-years in 2014^22^ and can be compared with another Canterbury study from 2004.^10^ Over the 10 years between the 2 studies, the incidence of IBD increased from 25.2 to 39.8. A follow-up study was also conducted in Barwon, Australia, over a shorter 2-year period.^12^ These studies from the same region observed relatively consistent incidence rates of 29.3^11^ and 24.2^12^ per 100 000 person-years.

Fourteen studies reported incidence rates for both CD and UC. Nine of these studies found higher rates of CD with CD:UC ratios of >1.^10-13,19,22-25^ Two population-based studies showed particularly high proportions of CD, with ratios above 2.^10,12^ The early research studies (pre-2000) showed higher proportions of UC with ratios of 0.5 or less.^29,30,32^ This may reflect shifts in technology over time, as increasing use of diagnostic imaging, capsule, and upper gastrointestinal endoscopy may increase diagnoses of CD. However, 2 recent studies described greater numbers of UC diagnoses with ratios of 0.8 (Supplementary Figure 2).^20,21^

### Characteristics of Incident Populations

The sex of incident cases of IBD was evenly split between males and females for 9^13,19-22,24,25,30,32^ of the 12 studies reporting this information (Supplementary Table 3). The remaining 3 studies^11,28,31^ showed a higher proportion of females. This pattern is consistent with other international studies^38^ and may be connected to the CD:UC ratios, as more severe CD is associated with females and more severe UC is associated with males.^38^ The mean and median ages of participants both ranged between 34 and 43 years.

The 8 studies^19,20,23,24,29,31-33^ reporting ethnicity all reported that the majority of participants were of European descent, which is the predominant ethnic group in New Zealand and Australia. The most recent study from Rotorua, New Zealand, collected data up to 2020 and reported the highest proportion of Māori (9.2%).^19^ The authors noted that almost 37% of the Rotorua’s general population are Māori, indicating that there is less IBD diagnosed in Māori.^19^ Other studies each found <5% Māori,^20,24,33^ and similarly low proportions of Pacific Islanders^29,31,32^ and indigenous Australians^23^ were observed.

Eleven studies^11-13,21,22,24,25,28,30-32^ described the disease pattern of the included participants in terms of location, behavior, or extent, with the recent studies using Montreal phenotypes (Supplementary Table 3).^39^ Inflammatory disease was the predominant CD behavior (78%-94%), and the proportions of perianal disease ranged from 9% to 23%. CD location and UC extent varied between the studies, with no particular category being predominant.

Three early studies^30-32^ reported varying surgery rates from 11% to 61%. In contrast, 2 more recent Australian studies reported 18% of patients with CD had a resection^25^ and 10% of patients had surgery within a year of diagnosis.^13^ This study also reported 19% of patients had a family history of IBD.^13^

### Pooled Incidence Rates

The pooled incidence rate for IBD was 19.8 per 100 000 person-years (**Table 2**). The pooled rate for CD (8.3) was higher than the pooled UC rate (7.4), with the IBDU pooled rate being much lower, at 0.7 per 100 000 person-years. There were high levels of heterogeneity (>80%) in each of the pooled estimates, reflecting the differences between the individual studies.

**Table 2.** Pooled incidence and prevalence rates and sensitivity analysis

	IBD			CD			UC			IBDU		
	Rate (95% CI)	*I* ^2^	n	Rate (95% CI)	*I* ^2^	n	Rate (95% CI)	*I* ^2^	n	Rate (95% CI)	*I* ^2^	n
Pooled incidence rate, per 100 000 person-years												
All data	19.8 (15.8-23.7)	99.1	15	8.3 (6.9-9.8)	98.9	17	7.4 (5.7-9.1)	96.7	15	0.7 (0.4 to 1.0)	80.1	11
Population-based studies	27.2 (22.1-32.3)	95.5	8	15.2 (11.4-19.0)	95.2	9	10.2 (8.6-11.8)	75.2	8	0.9 (0.5-1.2)	67.4	8
Hospital-based studies	11.3 (7.5-15.2)	99.1	7	2.8 (1.8-3.9)	98.1	8	4.2 (2.5-5.9)	95.9	7	0.5 (0.1-0.9)	92.0	3
Australian studies	23.5 (12.8-23.7)	98.4	8	7.7 (6.0-9.5)	98.8	9	9.4 (5.0-13.8)	97.5	7	1.1 (0.4-1.7)	83.4	6
NZ studies	18.3 (12.4-34.7)	99.1	6	9.2 (6.5-11.9)	99.0	8	6.9 (5.2-8.7)	96.0	7	0.6 (0.2-1.0)	79.6	5
Post-2000 studies	24.0 (19.3-28.8)	99.3	12	12.9 (10.3-15.6)	96.4	12	9.1 (7.1-11.2)	94.0	11	0.8 (0.5-1.2)	76.1	10

Pooled prevalence rate, per 100 000 persons												
All data	303.3 (128.1-478.4)	99.9	11	149.8 (71.0-228.5)	99.7	11	142.2 (63.1-221.4)	99.8	11	9.5 (5.8-13.2)	93.6	10
Population-based studies	337.3 (279.9-394.6)	98.8	7	168.4 (131.4-205.3)	98.1	7	155.4 (129.6-181.1)	96.7	7	9.8 (6.7-12.9)	92.5	6
Non–population-based studies	242.6 (0-633.0)	99.9	4	116.2 (0-290.8)	99.9	4	118.3 (0-301.2)	99.9	4	7.5 (0.5-14.5)	95.9	4
Australian studies	346.2 (138.1-554.3)	99.9	6	168.3 (73.7-263.0)	99.8	6	141.1 (30.0-252.2)	99.8	7	9.5 (4.7-14.3)	95.4	7
NZ studies	310.4 (225.2-395.6)	98.6	4	157.2 (116.1-198.3)	96.5	4	144.16 (87.2-201.1)	98.1	4	9.2 (5.7-12.6)	76.8	3

Pooled estimates were calculated for groups with more than 2 contributing studies.

Abbreviations: CD, Crohn’s disease; CI, confidence interval; IBD, inflammatory bowel disease; IBDU, inflammatory bowel disease unclassified; UC, ulcerative colitis; NZ, New Zealand.

## Prevalence Studies

**Figure 3.**
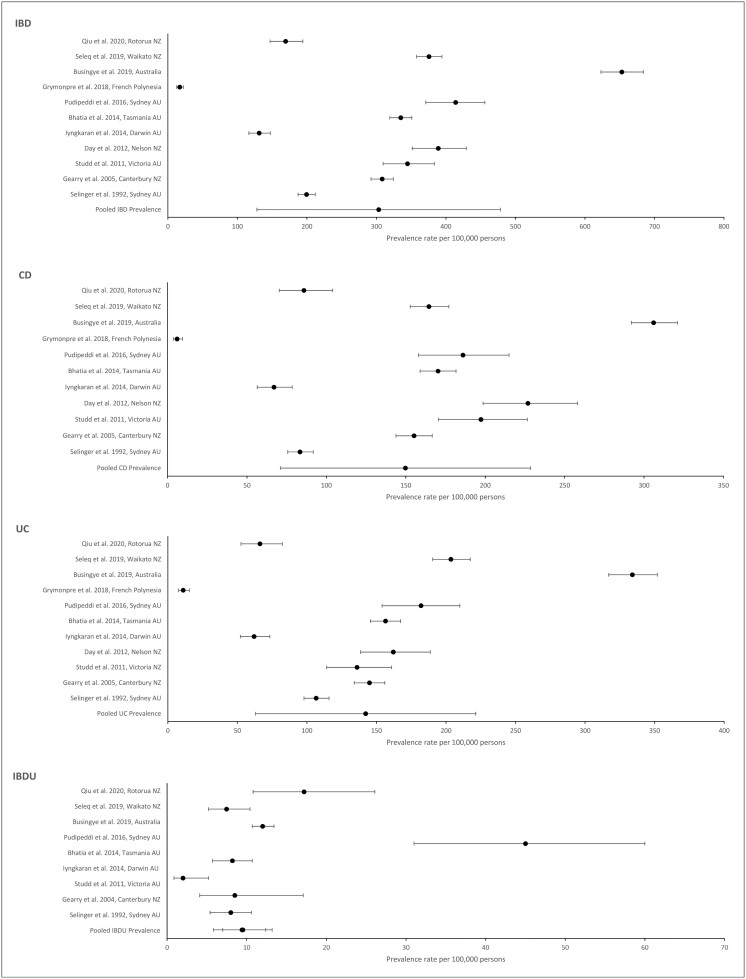
Inflammatory bowel disease (IBD), Crohn’s disease (CD), ulcerative colitis (UC), and IBD unclassified (IBDU) prevalence rates. AU, Australia; NZ, New Zealand.

Eleven studies^10,12,13,19,20,23,26,34-37^ reported the prevalence of IBD, CD, and UC, with 10 of these also providing IBDU prevalence rates. Six studies were from areas within Australia,^12,13,23,34,36,37^ 4 were from within New Zealand,^10,19,20,26^ and 1 was from French Polynesia (Figure 3).^35^

Seven studies used population-based data collection methods,^10,12,13,20,26,36,37^ 2 used hospital-based methods,^19,23,35^ and 1 used general practice (GP) records.^34^ This GP-based study, conducted in Australia in 2019, produced the highest reported prevalence rate of 653 per 100 000 persons for IBD.^34^ However, the quality assessment noted a risk of bias with this methodology. The lowest prevalence rate (17 per 100 000 persons) was from a hospital-based study conducted in 2018 in French Polynesia.^35^ The 2 other hospital-based studies also reported relatively low prevalence rates of 131.2^23^ and 169.3^19^ per 100 000 persons. Unlike the incidence studies, the more recent prevalence studies did not show higher prevalence rates compared with earlier studies.

The range of CD:UC ratios was narrow (0.6-1.5), with 7 studies having ratios above 1,^10,12,13,19,23,26,36^ indicating that CD was more prevalent, and 4 having values <1.^20,34,35,37^

### Characteristics of Prevalent Populations

For most prevalence studies, the proportion of males to females was even,^10,19,20,23,35-37^ but 2 studies reported slightly more females, at 57%^34^ and 58%.^13^ The mean and median ages ranged from 32 to 52 years, as some prevalence studies reported current age and others reported age at diagnosis. European/Caucasian was the predominant ethnicity, with Māori, Polynesian, and Indigenous Australian and Torres Strait Islanders making up <10% of each study reporting ethnicity information.^10,19,20,23,34,35^ The prevalent populations showed more complicated CD behaviors (stricturing and penetrating disease) compared with the incidence populations, reflecting differences in disease duration. Surgery rates ranged from 20% to 41%, and one study reported that 17% of patients had a family history of IBD.^10^

### Pooled Prevalence Rates

The pooled prevalence rate for IBD was 303.3 per 100 000 persons. The pooled rate for CD: 149.8 was very similar to that for UC (142.2), but a lower rate was seen for IBDU (9.5).

### Quality and Sensitivity Analysis

A sensitivity analysis calculated the pooled incidence and prevalence rates for the population-based studies, and these were higher than the all-studies estimates for each category. The IBD population-based pooled incidence was 27.2 per 100 000 person-years (CD 15.2, UC 10.2, and IBDU 0.9). For prevalence, the population-based pooled rates were 337.3 per 100 000 persons for IBD (168.4 per 100 000 persons for CD, 155.4 per 100 000 persons for UC, and 9.8 per 100 000 persons for IBDU) (Table 2). The pooled population-based studies also had slightly lower *I*^2^ values, indicating that a small proportion of the heterogeneity could be attributed to study design. In contrast, the hospital and non–population-based studies had lower pooled estimates and higher *I*^2^ values. The pooled estimates from the Australian and New Zealand studies varied without a clear pattern. The studies completed after the year 2000 had higher pooled incidence rates compared with the earlier studies. However, there were not enough prevalence studies conducted before 2000 to calculate pooled prevalence rates. Similarly, there were not enough incidence or prevalence studies from the Pacific Islands to calculate regional pooled estimates from those areas.

The quality assessment found that 23 studies had a low risk of misclassification bias as standard diagnostic criteria were utilized to identify and confirm cases of IBD (Supplementary Table 4). The risk of selection bias was rated low or very low for 24 studies, with 2 studies being rated as moderate risk.

## Discussion

This review showed high pooled incidence rates for IBD, CD and UC. These were driven primarily by recent population-based studies from Australia and New Zealand. Studies from these countries also led the high pooled prevalence rates observed. These data suggest that Australia and New Zealand are entrenched in the third epidemiological stage of compounding prevalence with high incidence and prevalence.

A 4-stage epidemiological model of IBD predicts countries go through a stage of increasing incidence as they develop economically and IBD is unmasked.^8^ This accelerated incidence stage is heightened by the young age at diagnosis and the low mortality rates associated with IBD, leading to rapidly increasing prevalence rates.^8^ The studies after 2000 (the year compounding prevalence was predicted to start for Western countries) reported a range of moderate-to-high incidence rates without a clear plateau. These recent studies were all from Australia and New Zealand, demonstrating progressively higher prevalence rates to support the concept of the Western countries of Oceania being in stage 3 (compounding prevalence). The 2019 Australian GP based study^34^ appears to be an outlier, with a prevalence rate over 200 people higher than the next highest study. This particularly high prevalence rate is likely the result of the different methodological approach used, which involved searching administrative diagnosis codes for IBD and synonyms of CD and UC.^34^

In contrast, the paucity of studies from countries other than Australia and New Zealand is a key finding and highlights an important gap in the worldwide study of IBD. The 2 Pacific Island studies that were included in the review, an incidence study from Fiji^29^ and a prevalence study from French Polynesia,^35^ both showed low rates of IBD. Other authors^40,41^ have recognized the value in studying IBD in developing countries and investigated the relationship between environmental factors such as urbanization and the incidence and prevalence of IBD.^6^ Although French Polynesia is not a developing country, Fiji is, and changes in a country’s economic status will likely lead to changes in the rate of IBD observed. Future studies are necessary to assess whether the Pacific Island nations in Oceania are transitioning from the first epidemiologic stage of emergence to the second of accelerated incidence. Prospective population-based studies in different locations, with special attention paid to indigenous groups, would address this knowledge gap.

In addition, studies from Western countries in this review each noted low proportions of Māori,^10,20,24,33^ Pacific Islanders,^31,32^ and Indigenous Australians^23,34^ with IBD. A recent study from the North Island of New Zealand^19^ also reported relatively low rates of IBD in Māori over its 20-year study period but postulated that rates are increasing. The rising incidence of IBD in Māori is likely to be multifactorial including inequities in access to healthcare, differences genetic susceptibility, and evolving environmental influences such as smoking, household crowding, and diet.^19^ The higher background population of Māori in the North Island of New Zealand is also likely to influence the rates of IBD observed and reinforces the importance of studying IBD epidemiology in different contexts. Across a country with a large land area like Australia, different geographical regions may have distinct population characteristics and differences in economic development. Within Australia and New Zealand there were several areas without any studies: these included the state of Western Australia and Northland in New Zealand, which is an area with a high proportion of Māori.

The CD:UC ratios observed in the current review (earlier studies with more UC and the majority of recent studies showing more CD) support the theory that UC is more common until healthcare infrastructure and technology develop sufficiently to allow the more nuanced diagnosis of CD.^8,42^ The specific criteria used to discriminate between CD and UC were not provided in most of the epidemiological studies in this review, but changes in criteria over time may also impact the ratios observed. Furthermore, both Pacific Island studies reported a predominance of UC.

Smoking has a long association with IBD, and although information on smoking was not collected in this study, it is also possible that changes in the rates of smoking may also influence the CD:UC ratios observed.

It is clear that increasing rates of IBD come with increasing costs to the health system in terms of specialist care, medications, surgery, and hospitalizations.^1^ Certain disease characteristics are associated with a more severe disease course and, therefore, indicate a particularly high burden on the health system. Several studies in our review reported disease phenotypes, the more recent of these using the Montreal classification.^39^ It was noted the incident cohorts reported high proportions of inflammatory disease behavior,^11-13,21,22,25^ whereas the prevalence cohorts displayed relatively more of the complicated behaviors.^10,19,36^ This aligns with the current understanding of IBD as a progressive disease with the cumulative risk of developing complications (and the resulting need for surgery) increasing with time since diagnosis.^43-45^ The preponderance of CD seen in the current review may also be considered a signal of increased future costs, as unlike with UC, total colectomy is not considered curative.^46^ Age at diagnosis, onset in childhood, and especially very early onset IBD are recognized risk factors for a more severe disease course.^44^ This study did not include pediatric-specific cohorts, but a recent systematic review and meta-analysis reported high levels of pediatric IBD in Oceania.^17^

A strength of this review is the broad inclusion criteria, which allowed a full examination of 30 studies from Oceania. One of the included Pacific Island studies was older, from 1985 to 1986,^29^ and both Pacific studies used hospital-based data collection.^29,35^ Other international systematic reviews have focused on the highest-quality population-based studies,^5,14^ and the current sensitivity analysis did show higher pooled incidence and prevalence rates when looking only at these studies. Nonetheless, if the review was restricted to these studies, there would be no information at all from the Pacific Islands. Another strength of this study is the meta-analysis, which enabled the production of the first pooled estimates for IBD, CD, UC, and IBDU for the region. The analysis of both incidence and prevalence allowed the consideration of Oceania in terms of the epidemiological development model of IBD.

The comparison of crude incidence and prevalence rates is a limitation of the current study. Age-standardized rates remove the impact of differences in population structure, and although this made almost no difference in studies reporting this information from New Zealand (IBD incidence: crude = 39.8, age-standardized = 39.5)^22^ and Australia (IBD incidence: crude = 29.0, age-standardized = 29.5),^13^ it has the potential to impact developing countries. Another limitation is the high levels of heterogeneity observed in the pooled estimates. This was not unexpected due to the small number of studies, the different methodologies, differing population structures, and the differing study years included. Unfortunately, the small number of studies did not allow a robust assessment of the sources of heterogeneity. Time trends in incidence rates were also not examined in this review due to the small number of studies at each time period, but future work with more studies would contribute to our understanding and potentially support incidence predictions.

## Conclusions

This systematic review and meta-analysis provides the first pooled estimates of IBD incidence and prevalence in Oceania. The small number of Pacific Island studies highlights this area as an important gap in the understanding of global IBD epidemiology.

## Supplementary data

Supplementary data is available at *Inflammatory Bowel Diseases* online.

izad295_suppl_Supplementary_Material
