# Supplementary material for: The Epidemiology of Inflammatory Bowel Disease in Oceania: A Systematic Review and Meta-Analysis of Incidence and Prevalence
Source: Inflamm Bowel Dis. 2023 Dec 30;30(11):2076–86. doi: 10.1093/ibd/izad295 (PMC11532596; doi:10.1093/ibd/izad295)
Supplement: izad295_suppl_Supplementary_Material [file izad295_suppl_supplementary_material.docx]

**Supplementary Data Content:**

**Table 1.** *Search Strategy*

**Table 2.** *Countries of Oceania*

**Figure 1:** *Incidence and Prevalence Rates by Year*

**Figure 2**: *CD:UC ratios for Incidence and Prevalence Studies*

**Table 3:** *Additional Study Characteristics*

**Table 4**: *Quality Assessment and Risk of Bias*

**Table 1.** *Search Strategy*

| **Database:** Ovid MEDLINE(R) ALL <1946 to April 10 2023>   \| 1 \| (Crohn* OR colitis OR IBD OR inflammatory bowel disease).mp. \| \| --- \| --- \| \| 2 \| (epidemiolog* OR inciden* OR prevalen*).mp. \| \| 3 \| (zealand* OR australia* OR australasia* OR pacific OR oceania OR polynesia* OR micronesia* OR melanesia* OR fiji* OR samoa* OR tonga* OR papua OR pasifika OR pasefika).mp. \| \| 4 \| 1 and 2 and 3 \| |
| --- | --- | --- | --- | --- | --- | --- | --- | --- |
| **Database:** Ovid EMBASE - All years <1947-Present with Daily Update>   \| 1 \| (IBD or inflammatory bowel disease* or Crohn* or colitis).mp. \| \| --- \| --- \| \| 2 \| (epidemiolog* or incidence or prevalence).mp. \| \| 3 \| (Oceania or Zealand or Australia or Australasia or pacific).mp. \| \| 5 \| 1 and 2 and 3 \| |
| **Database:** Elsevier Scopus <1970 to present>   \| 1 \| (Crohn* or colitis or IBD or (inflammatory and bowel and disease) TITLE-ABS-KEY \| \| --- \| --- \| \| 2 \| (epidemiolog* or inciden* or prevalen*) TITLE-ABS-KEY \| \| 3 \| (zealand* OR australia* OR australasia* OR pacific OR oceania OR polynesia* OR micronesia* OR melanesia* OR fiji* OR samoa* OR tonga* OR papua OR pasifika) TITLE-ABS-KEY \| \| 4 \| 1 and 2 and 3 \|   **Database:** Clarivate Web of Science -Core Collection <April 11 2023>   \| 1 \| Crohn* OR colitis OR “inflammatory bowel disease” (All Fields) \| \| --- \| --- \| \| 2 \| epidemiolog* OR inciden* OR prevalen* (All Fields) \| \| 3 \| zealand* OR australia* OR australasia* OR pacific OR oceania OR polynesia* OR micronesia* OR melanesia* OR fiji* OR samoa* OR tonga* OR papua OR pasifika OR pasefika (All Fields) \| \| 4 \| 1 and 2 and 3 \| |

**Table 2.** *Countries of Oceania*

| **Region Name** | **Sub-region Name** | **Country or Area** |
| --- | --- | --- |
| Oceania | Australia and New Zealand | Australia |
| Oceania | Australia and New Zealand | Christmas Island |
| Oceania | Australia and New Zealand | Cocos (Keeling) Islands |
| Oceania | Australia and New Zealand | Heard Island and McDonald Islands |
| Oceania | Australia and New Zealand | New Zealand |
| Oceania | Australia and New Zealand | Norfolk Island |
| Oceania | Melanesia | Fiji |
| Oceania | Melanesia | New Caledonia |
| Oceania | Melanesia | Papua New Guinea |
| Oceania | Melanesia | Solomon Islands |
| Oceania | Melanesia | Vanuatu |
| Oceania | Micronesia | Guam |
| Oceania | Micronesia | Kiribati |
| Oceania | Micronesia | Marshall Islands |
| Oceania | Micronesia | Micronesia (Federated States of) |
| Oceania | Micronesia | Nauru |
| Oceania | Micronesia | Northern Mariana Islands |
| Oceania | Micronesia | Palau |
| Oceania | Micronesia | United States Minor Outlying Islands |
| Oceania | Polynesia | American Samoa |
| Oceania | Polynesia | Cook Islands |
| Oceania | Polynesia | French Polynesia |
| Oceania | Polynesia | Niue |
| Oceania | Polynesia | Pitcairn |
| Oceania | Polynesia | Samoa |
| Oceania | Polynesia | Tokelau |
| Oceania | Polynesia | Tonga |
| Oceania | Polynesia | Tuvalu |
| Oceania | Polynesia | Wallis and Futuna Islands |

Source: United Nations Statistical Division table M49: as at 25 June 2022

**Figure 1:** *Incidence and Prevalence Rates by Year*

*
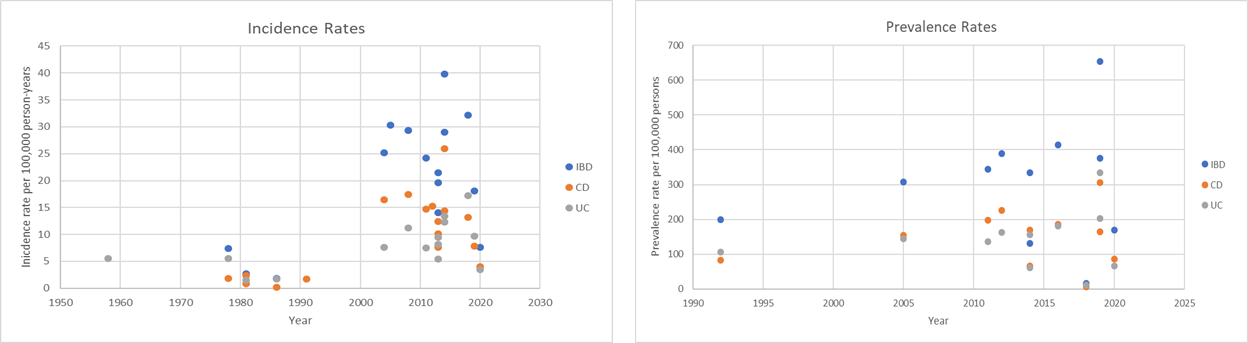
*

**Figure 2**: *CD:UC ratios for Incidence and Prevalence Studies*


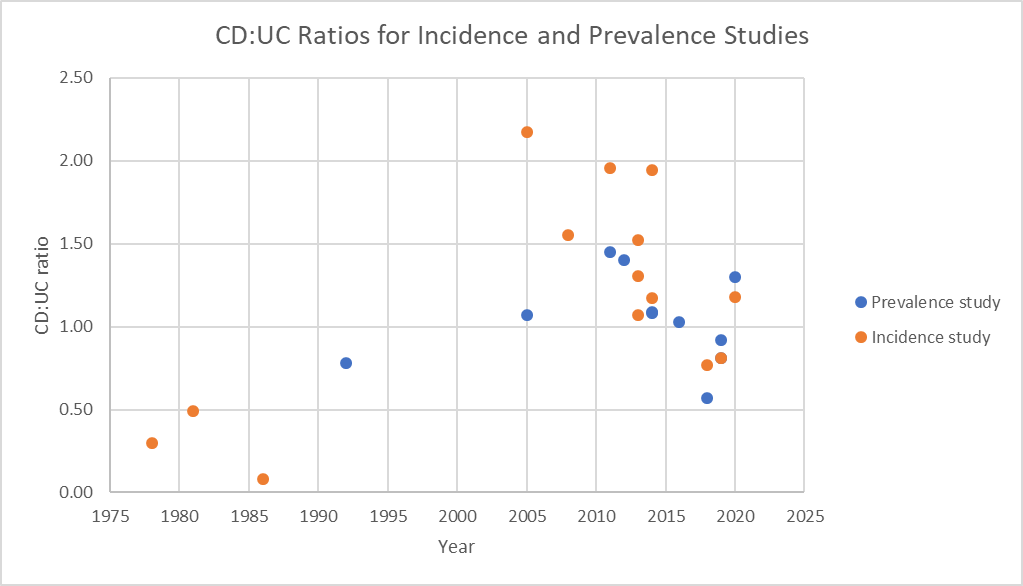


**Table 3:** *Additional Study Characteristics*

| **Author, Publication Year** | **Study Location, Date** | **Study Type** | **Disease** | **N (%)** | **Sex** | **Age** | **Ethnicity** | **Phenotype** | **Surgery** | **Family History** |
| --- | --- | --- | --- | --- | --- | --- | --- | --- | --- | --- |
| Qiu *et al*, 2022 ^19^ | New Zealand Rotorua,  2001-2020 | Incidence | IBD  CD  UC  IBDU | *152*  *80 (53%)*  *68 (45%)*  *4 (3%)* | *51%* Male,  *49%* Female | ND | *9.2%* Māori,  *91% European and other* | ND | ND | ND |
| Seleq *et al*, 2023 ^20^ | New Zealand Waikato, 2010-2019 | Incidence | IBD  CD  UC  IBDU | 710  309 *(44%)*  380 *(54%)*  21 *(2%)* | 51% Male,  49% Female | Mean age at diagnosis 39.3 | 4.5% Māori 83.0% European | ND | ND | ND |
| Flanagan *et al*, 2022 ^21^ | Australia Mackay, Isaac, Whitsunday, 2017-2018 | Incidence | IBD  CD  UC  IBDU | 56  23 *(41%)*  30 *(54%)*  3 *(5%)* | 50% Male, 50% Female | Median age at diagnosis 36 | ND | CD: L1 39%, L2 13%, L3 48%, L4 39%. 9% perianal disease. B1 78%, B2 13%, B3 17%.  UC: E1 30%, E2 17% E3 53%. | ND | ND |
| Su *et al*, 2016 ^22^ | New Zealand Canterbury, 2014 | Incidence | IBD  CD  UC  IBDU | 205  134 (65%)  69 (34%)  2 (1%) | 49% Male, 51% Female | CD median 36 years, UC median 43 years | ND | CD: L1 29%, L2 35%, L3 32%, L4 22%. B1 94%, B2 3.8%, B3 2.3% B3. 13.5% perianal.  UC: E1 30%, E2 32%, E3 38%. | ND | ND |
| Bhatia *et al*, 2019 ^13^ | Australia Tasmania,  2013-2014 | Incidence | IBD  CD  UC  IBDU | 149  74 (50%)  63 (42%)  12 (8%) | 56% Male, 44% Female | Median age at diagnosis 41 years (IQR 34) | ND | CD: L1 28%, L2 37% L3 34%. B1 81%, B2 15%, B3 4%.  UC: E1 32%, E2 32%, E3 37%. | 13% had surgery within a year of diagnosis | 30% of CD and 10% of UC had a family history of IBD |
| Iyngkaran *et al*, 2015 ^23^ | Australia Darwin,  2013 | Incidence | IBD  CD  UC | 29  15 (*52*%)  14 (*48*%) | ND | ND | *3*% Indigenous Australian | ND | ND | ND |
| Coppell *et al*, 2018 ^24^ | New Zealand Otago,  1996-2013 | Incidence | IBD  CD  UC  IBDU | 453  236 (52%)  181 (40%)  36 (8%) | 51% Male, 49% Female | Median age 34 years | New Zealand European 97%, Māori 1.8% | CD: L1 24%, L2 32%, L3 44%. Perianal disease 14%. | ND | ND |
| Niewiadomski *et al*, 2015 ^25^ | Australia Barwon Victoria,  2010-2013  2007-2008 | Incidence | IBD  CD  UC  IBDU | 252  146 (58%)  96 (38%)  10 (4%) | 45% Male, 55% Female | Median age at diagnosis 36 years (CD) | ND | CD: L1 32%, L2 30%, L3 38%, L4 12%. B1 80%, B2 10%, B3 10%. Perianal disease 12%.  UC: E1 32%, E2 31%, E3 36%. | 18% of CD patients, 6% of UC patients |  |
| Day *et al,* 2014 ^26^ | New Zealand Nelson, 2012 | Incidence | CD | 15 | ND | ND | ND | ND | ND | ND |
| Studd *et al,* 2016 ^12^ | Australia, Barwon 2010-2011 | Incidence | IBD  CD  UC  IBDU | 71  43 (*61%)*  22 (*31%)*  6 (*8%)* | ND | Median age 38 years | ND | CD: L1 30%, L2 26%, L3 44%, L4 7%. Perianal disease 14%.B1 88%, B2 9.3%, B3 2.3%  UC: E1 32%, E2 27% E3 41%. | ND | ND |
| Wilson *et al*, 2010 ^11^ | Australia, Barwon  2007-2008 | Incidence | IBD  CD  UC  IBDU | 76  45 (59%)  29 (38%)  2 (3%) | *43%* Male,  *57%* Female | Median age 34 years (CD) | ND | CD: L1 49%, L2 27%, L3 24%. B1 93%, B2 4.5%, B3 2.5%  UC: E1 35%, E2 48%, E3 17%. | ND | ND |
| Hanigan *et al,* 2008 ^27^ | Australia, North Brisbane 2005 | Incidence | IBD | 222 | ND | ND | ND | ND | ND | ND |
| Gearry *et al*, 2006 ^10^ | Canterbury, New Zealand, 2004 | Incidence | IBD  CD  UC  IBDU | 116  76 (66%)  35 (30%)  5 (4%) | ND | ND | ND | ND | ND | ND |
| Anseline *et al*, 1995 ^28^ | Australia, Hunter Valley  1967-1991 | Incidence | CD | 130 | 42% Male, 58% Female | ND | ND | 35% small bowel disease, 28% colonic disease, 32% ileocolonic disease, 4.6% disease in other locations. | 100% (surgical cohort) | ND |
| Probert *et al,* 1991 ^29^ | Fiji,  1985-1986 | Incidence | IBD  CD  UC | 13  1 (*8%)*  12 (*92%)* | ND | ND | *87*% Fijian Indians, *7%* Melanesian, *7*% other | ND | ND | ND |
| McDermott *et al*, 1987 ^30^ | Australia Melbourne,  1980-1981 | Incidence | IBD  CD  UC  IBDU | 81  24 (*30%)*  49 (*60%)*  8 (*10%)* | 49% Male,  51% Female | ND | ND | CD: 23% small bowel disease, 35% large bowel disease, 43% small and large bowel disease.  UC: 22% rectal disease. | 20% of CD patients had a resection | ND |
| Schlup *et al*, 1986 ^31^ | New Zealand Dunedin,  1972-1981 | Incidence | CD | 48 | 38% Male, 62% Female | Mean age 39.5 years | No Polynesian participants | CD: 23% small bowel disease, 60% large bowel disease, 17% large bowel and terminal ileum. Perianal disease 23%. | 44% required surgery | ND |
| Eason *et al*, 1982 ^32^ | New Zealand, Auckland  1969-1978 | Incidence | IBD  CD  UC | 593  137 (*23%)*  456 (*77*%) | 51% Male, 49% Female | Mean age 37-38 years | 0.3% Polynesian | CD: 18% small bowel, 26% colonic, ileocolic 52%, anorectal 4%.  UC: 32% distal, 35% substantial, 33% universal. | 61% of CD patients had a resection, 23% of UC patients | ND |
| Wigley *et al*, 1962 ^33^ | New Zealand, Wellington  1954-1958 | Incidence | UC | 51 | ND | ND | 4% Māori | ND | ND | ND |
| Qiu *et al,* 2022 ^19^ | New Zealand Rotorua,  2020 | Prevalence | IBD  CD  UC  IBDU | 197  100 (51%)  77 (39%)  20 (10%) | 51% Male,  49% Female | Median age 48 years | 7.6% Māori,  92.4% European/other | CD: L1 15%, L2 17%, L3 60%, L4 11%.  B1 62%, B2 30%, B3 8%.  UC: E1 19%, E2 36%, E3 45%. | 25% had surgery for IBD | ND |
| Seleq *et al,* 2023 ^20^ | New Zealand Waikato, 2019 | Prevalence | IBD  CD  UC  IBDU | 1611  706 (44%)  873 (54%)  32 (2%) | 47% Male,  53% Female | Mean current age 50.4 years | 3.7% Māori, 22.1% other,  74.2% NZ European | CD: L1 25%, L2 33%, L3 36%. Perianal disease 13%.  UC: E1 22%, E2 34%, E3 37%. | ND | ND |
| Busingye *et al,* 2021 ^34^ | Australia, 2019 | Prevalence | IBD  CD  UC  IBDU | 15859  7442 *(47%)*  8121 *(51%)*  296 *(1.9%)* | 43% Male, 57% Female | Median current age 52 years | 1.6 % Aboriginal and/or Torres Strait Islander, 82% other | ND | ND | ND |
| Grymonpre *et al*, 2019 ^35^ | French Polynesia, 2018 | Prevalence | IBD  CD  UC  IBDU | 49  17 (*35%)*  30 (*61%)*  2 (*4%)* | 51% Male, 49% Female | Median age at diagnosis 43 years | 72% Caucasian, 10% Polynesian | ND | ND | ND |
| Pudipeddi *et al*, 2021 ^36^ | Australia Sydney,  2016 | Prevalence | IBD  CD  UC  IBDU | 364  164 (45%)  160 (44%)  40 (11%) | 49% Male, 51% Female | Median age 47 years | ND | CD: L1 26%, L2 32%, L3 41%. B1 60%, B2 21%, B3 19%. 23% perianal disease.  UC: E1 35%, E2 39%, E3 25%. | ND | ND |
| Bhatia *et al*, 2019 ^13^ | Australia Tasmania,  2014 | Prevalence | IBD  CD  UC  IBDU | 1719  874 (51%)  803 (47%)  42 (2%) | 42% Male, 58% Female | Median age at diagnosis 36 years | ND | ND | 41% of CD had a resection and 7% of UC had a total colectomy | ND |
| Iyngkaran *et al*, 2015 ^23^ | Australia Darwin,  2014 | Prevalence | IBD  CD  UC  IBDU | 279  142 (51%)  132 (47%)  5 (2%) | 53% Male, 47% Female | Mean age at diagnosis 32.3 years | 88% Caucasian, Indigenous Australians 1% | ND | 20% had previous surgery | ND |
| Day *et al,* 2014 ^26^ | New Zealand Nelson, 2012 | Prevalence | IBD  CD  UC | *384*  224 (*58%)*  160 (*42%)* | ND | ND | ND | ND | ND | ND |
| Studd *et al,* 2016 ^12^ | Australia, Barwon | Prevalence | IBD  CD  UC  IBDU | 1011  579 (57%)  399 (39%)  25 (2%) | ND | Median age 46 years | ND | CD: L1 29%, L2 40%, L3 30%, L4 7%. Perianal disease 13%.  UC: E1 33%, E2 33%, E3 33%. | ND | ND |
| Gearry *et al*, 2006 ^10^ | New Zealand Canterbury, 2005 | Prevalence | IBD  CD  UC  IBDU | 1420  715 (50%)  668 (47%)  37 (3%) | 46% Male, 54% Female | Mean 47 years | Māori 1%  Other 99% | CD: L1 30%, L2 41%, L3 24%, L4 5%  B1 53%, B2 26%, B3 21%.  UC: E1 38%, E2 25%, E3 37%. | 25% had one or more resections | 17% first degree relative with IBD |
| Selinger *et al*, 2013 ^37^ | Australia, Sydney | Prevalence | IBD  CD  UC  IBDU | 997  417 (*42%)*  533 (*53%)*  47 (*5%)* | 48% Male,  52% Female | Median age at diagnosis 29 years (CD) | ND | ND | 27% required abdominal surgery | ND |

IBD- inflammatory bowel disease, CD-Crohn’s disease, UC-ulcerative colitis, IBDU-inflammatory bowel disease unclassified, GI-gastrointestinal tract. ND- not described. Montreal phenotype: CD: L1=ileal, L2=colonic, L3=ileocolonic, L4=isolated upper gastrointestinal involvement. B1=non-stricturing and non-penetrating, B2=stricturing, B3=penetrating. UC: E1=proctitis, E2=left-sided colitis, E3=pancolitis.

*Italics* -data derived from supporting information by the current authors. Rates are crude average annual incidence and prevalence per 100,000 person-years or per 100,000 of population.

**Table 4**: *Quality Assessment and Risk of Bias*

| **First Author, Publication Year** | **Study Location, Date** | **Length** | **Study Design** | **Case Ascertainment (risk of selection bias)** | **Case Identification (risk of misclassification bias)** | **Potential study overlaps** | **Information source** |
| --- | --- | --- | --- | --- | --- | --- | --- |
| Qiu *et al,* 2022 ^19^ | New Zealand Rotorua,  2001-2020 | 20 years | Retrospective, Hospital-based,  Incidence | Patients treated at public hospitals in the area (low) | Standard diagnostic criteria (low) | - | Full journal article |
| Seleq *et al*, 2023 ^20^ | New Zealand Waikato, 2010-2019 | 10 years | Retrospective, Population-based Incidence | Patients treated at all centres in the area (low) | Standard diagnostic criteria (low) | - | Full journal article |
| Flanagan *et al,* 2022 ^21^ | Australia Mackay, Isaac, Whitsunday, 2017-2018 | 1 year | Prospective, Population-based Incidence | Patients treated at all centres in the area (low) | Standard diagnostic criteria (low) | - | Full journal article |
| Su *et al*, 2016 ^22^ | New Zealand Canterbury, 2014 | 1 year | Prospective  Population-based  Incidence | Patients treated at all centres in the area (very low) | Standard diagnostic criteria (low) | - | Full journal article |
| Bhatia *et al*, 2019 ^13^ | Australia Tasmania,  2014 | 1 year | Prospective  Population-based  Incidence | Patients treated at all centres in the state (very low) | Standard diagnostic criteria (low) | - | Full journal article |
| Iyngkaran *et al*, 2015 ^23^ | Australia Darwin,  2013 | 1 year | Retrospective Hospital-based Incidence | Patients treated at the only public tertiary referral centre in the state (low) | ND | - | Conference Abstract and PhD thesis |
| Coppell *et al*, 2018 ^24^ | New Zealand Otago, 1996-2013 | 18 years | Retrospective Hospital-based Incidence | Patients treated at the only public tertiary referral centre in the region (low) | Standard diagnostic criteria (low) | - | Full journal article |
| Niewiadomski *et al,* 2015 ^25^ | Australia Barwon Victoria, 2007-2008, 2010-2013 | 4 years | Prospective  Population-based  Incidence | Patients treated at all centres in the area (low) | Standard diagnostic criteria (low) | Risk of overlap with the other studies from Victoria: Wilson *et al*, Studd *et al*. | Full journal article |
| Day *et al*, 2014 ^26^ | New Zealand Nelson,  2012 | 1 year | Prospective  Population-based  Incidence | ND | ND | - | Full journal article focused on children |
| Studd *et al,* 2016 ^12^ | Australia Barwon, 2011 | 1 year | Prospective  Population-based  Incidence | Patients treated at all centres in the area (very low) | Standard diagnostic criteria (low) | Risks of overlap with other Victoria study Niewiadomski *et al*. | Full journal article |
| Wilson *et al*, 2010 ^11^ | Australia Barwon,  2007-2008 | 1 year | Prospective  Population-based  Incidence | Patients treated at all centres in the area (very low) | Standard diagnostic criteria (low) | Risks of overlap with other Victoria study Niewiadomski *et al.* | Full journal article |
| Hanigan *et al*, 2008 ^27^ | Australia North Brisbane, 2005 | 1 Year | Retrospective,  Hospital-based, Incidence | ND | ND | - | Conference Abstract |
| Gearry *et al*, 2006 ^10^ | New Zealand Canterbury, 2004 | 1 year | Prospective  Population-based Incidence | Patients treated at all centres in the area (very low) | Standard diagnostic criteria (low) | - | Full journal article and PhD thesis |
| Anseline *et al*, 1995 ^28^ | Australia, Hunter Valley | 21 years | Retrospective Hospital-based Incidence | Patients treated surgically in the main referral centre in the area (moderate) | Standard diagnostic criteria and histological confirmation after surgery (low) | - | Full journal article |
| Probert *et al*, 1991 ^29^ | Fiji,  1985-1986 | 2 years | Retrospective Hospital-based Incidence | Patients treated in all centres in the country with public health records (low) | ICD9 codes (moderate) | - | Full journal article |
| McDermott *et al*, 1987 ^30^ | Australia Melbourne,  1980-1981 | 1 year | Prospective Hospital-based  Incidence | Patients treated in 6 public hospitals in the area (low) | Standard diagnostic criteria (low) | - | Full journal article |
| Schlup *et al*, 1986 ^31^ | New Zealand Dunedin,  1972-1981 | 10 years | Retrospective Hospital-based Incidence | Patients treated at the only public tertiary referral centre in the area (low) | Standard diagnostic criteria (low) | - | Full journal article |
| Eason *et al*, 1982 ^32^ | New Zealand Auckland,  1969-1978 | 10 years | Retrospective Hospital-based Incidence | Patients treated at all the public hospitals in the area (low) | Standard diagnostic criteria (low) | - | Full journal article |
| Wigley *et al*, 1962 ^33^ | New Zealand, Wellington | 5 years | Retrospective Hospital-based Incidence | Patients treated at all the public hospitals in the area (low) | Standard diagnostic criteria (low) | - | Full journal article |
| Qiu *et al*, 2022 ^19^ | New Zealand Rotorua,  2001-2020 | 1 year | Retrospective, Hospital-based,  Prevalence | Patients treated at the public hospital in the area (low) | Standard diagnostic criteria (low) | - | Full journal article |
| Seleq *et al,* 2023 ^20^ | New Zealand Waikato, 2019 | 1 year | Retrospective, Population-based, Prevalence | Patients treated at all centres in the area (low) | Standard diagnostic criteria (low) | - | Full journal article |
| Busingye *et al,* 2021 ^34^ | Australia, 2019 | 1 year | Retrospective, General Practice records-based, Prevalence | Patients treated by General Practitioners (moderate) | IBD terms and synonyms from a database (high) | - | Full journal article |
| Grymonpre *et al*, 2019 ^35^ | French Polynesia, 2018 | 1 year | Retrospective, Hospital-based,  Prevalence | Patients treated at all centres in the area (low) | Standard diagnostic criteria (low) | - | Conference Abstract |
| Pudipeddi *et al*, 2021 ^36^ | Australia Sydney,  2016 | 1 year | Prospective, Population-based, Prevalence | Patients treated at one centre in the area (moderate) | Standard diagnostic criteria (low) | - | Full journal article |
| Bhatia *et al*, 2019 ^13^ | Australia Tasmania,  2014 | 1 year | Prospective  Population-based  Prevalence | Patients treated at all centres in the state (very low) | Standard diagnostic criteria (low) | - | Full journal article |
| Iyngkaran *et al*, 2015 ^23^ | Australia Darwin,  2014 | 1 year | Retrospective Hospital-based Prevalence | Patients treated at the only public tertiary referral centre in the state (low) | ND | - | Conference Abstract and PhD thesis |
| Day *et al*, 2014 ^26^ | New Zealand Nelson,  2012 | 1 year | Prospective  Population-based  Prevalence | ND | ND | - | Full journal article focused on children |
| Studd *et al,* 2016 ^12^ | Australia Barwon, 2011 | 1 year | Prospective, Population-based, Prevalence | Patients treated at all centres in the area (very low) | Standard diagnostic criteria (low) | - | Full journal article |
| Gearry *et al*, 2006^10^ | New Zealand Canterbury, 2005 | 1 year | Prospective  Population-based Prevalence | Patients treated at all centres in the area (very low) | Standard diagnostic criteria (low) | - | Full journal article and PhD thesis |
| Selinger *et al*, 2013 ^37^ | Australia, Sydney | 1 year | Retrospective  Population-based Prevalence | Patients treated at all centres in the area (low) | Standard diagnostic criteria (low) | - | Full journal article |

ND-Not described, Niewiadomski *et al,* data was collected prospectively 2010-2013 and retrospective comparison 2007-2008.
